# Prevalence of *Stenotrophomonas maltophilia* colonization among patients receiving mechanical ventilation in long-term care facilities in Maryland, United States

**DOI:** 10.1128/jcm.01629-25

**Published:** 2026-03-11

**Authors:** Alexis L. Green, Anthony D. Harris, Ashley Heller, Elisabeth Vaeth, Lisa Pineles, J. Kristie Johnson

**Affiliations:** 1 Duke University Medical Center, Durham, North Carolina, USA; 2 University of Maryland School of Medicine 12264, Baltimore, Maryland, USA; 3 Institute for Health Computing, North Bethesda, Maryland, USA; 4 Maryland Department of Health 1496, Baltimore, Maryland, USA; Maine Medical Center Department of Medicine, Portland, Maine, USA

**Keywords:** long-term care facilities, mechanically ventilated, *Stenotrophomonas maltophilia*

## Abstract

There is increasing concern regarding the importance of *Stenotrophomonas maltophilia* as a nosocomial infection, given its inherent antibiotic resistance, particularly for patients who have risk factors such as being mechanically ventilated. The prevalence of colonization receiving mechanical ventilation in chronic care facilities has not been studied. The Multi-Drug-Resistant Organism (MDRO) Prevention Collaborative performed a point prevalence of patients receiving mechanical ventilation in all 18 chronic care facilities in Maryland between 7 March 2023 and 8 June 2023. Patients had at least one sputum, skin, or perianal surveillance culture collected (579 samples). Samples were tested using three microbiological methods: (i) growth on Acinetobacter CHROMagar, (ii) growth on MacConkey agar with an imipenem disk, and (iii) growth in brain-heart infusion broth with an imipenem disk, followed by growth on MacConkey agar. 21.4% (124/579) of the samples collected grew *S. maltophilia*. Among 200 patients, 58.5% had at least one positive site (117/200). *S. maltophilia* was isolated from 58.2% (113/194) of sputum, 4.1% (8/193) of skin, and 1.6% (3/185) of perianal samples. Two patients had multiple samples that grew *S. maltophilia* for a total of 119 positive samples from 117 patients. Mechanically ventilated patients in chronic care facilities may be at risk of *S. maltophilia* colonization and potential infection.

**IMPORTANCE:**

There is increasing concern regarding the importance of *Stenotrophomonas maltophilia* as a nosocomial infection, given its inherent antibiotic resistance. *S. maltophilia* is widely present in the environment, and while not always virulent, it has been documented to have high morbidity and mortality among certain at-risk patient populations. We analyzed mechanically ventilated patients in chronic care facilities in the state of Maryland to quantify the burden of *S. maltophilia* in this population. More than half of patients receiving mechanical ventilation in chronic care facilities were colonized with *S. maltophilia,* which was most frequently isolated from sputum samples.

## INTRODUCTION

There is increasing concern regarding the importance of *Stenotrophomonas maltophilia* as a nosocomial infection risk, given its inherent antibiotic resistance. *S. maltophilia* has resistance genes and mutations that confer resistance to most β-lactams, including penicillins, cephalosporins, and carbapenem, as well as aminoglycosides. Beyond this, *S. maltophilia* can acquire efflux pumps that reduce the activity of trimethoprim/sulfamethoxazole, some tetracyclines, and fluoroquinolones (1). *S. maltophilia* is widely present in the environment, and while not always virulent, it has been documented to have high morbidity and mortality among certain at-risk patient populations (2, 3). As the use of life-saving, invasive medical interventions rises, so too does the reported prevalence of *S. maltophilia* colonization and infection (4). Multiple outbreaks of *S. maltophilia* have been documented in intensive care unit settings where mechanical ventilation is common, suggesting lapses in infection control accounting for the outbreaks (5–7). Recently, *S. maltophilia* pneumonia has been documented in patients with COVID-19 in the ICU and has been associated with longer hospital stays in this population (8, 9).

Prior colonization is an important risk factor for invasive disease by many respiratory pathogens (10), but the prevalence of colonization of *S. maltophilia* in adults receiving mechanical ventilation in chronic care facilities has not been studied. Furthermore, there are few studies comparing microbiological methods for isolating *S. maltophilia* for surveillance. This study utilized data from a 2023 statewide point prevalence survey to estimate the prevalence of *S. maltophilia* among patients receiving mechanical ventilation in chronic care facilities in the state of Maryland and compared three different microbiological methods for isolation (11).

## MATERIALS AND METHODS

### Data collection

**Fig 1:**
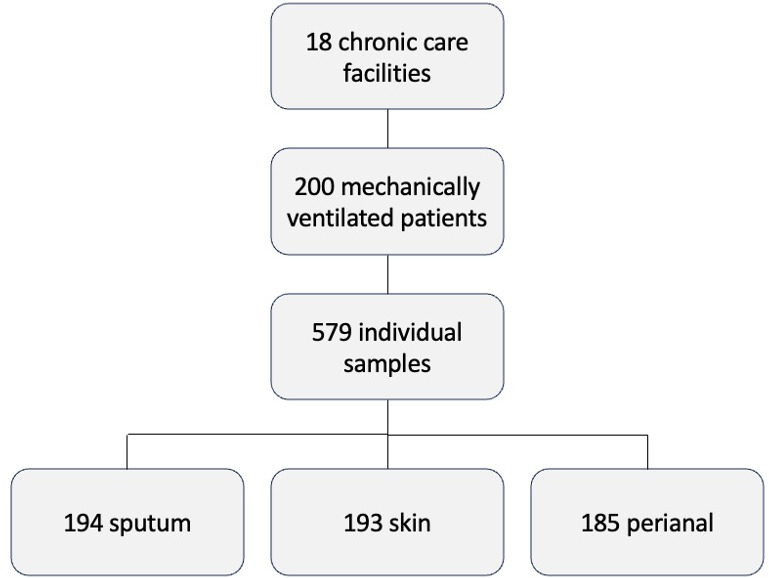
Overview of samples included in the study.

The Maryland Department of Health (MDH) Multi-Drug-Resistant Organism (MDRO) Prevention Collaborative has led multiple statewide point prevalence surveys to support their work to detect and prevent MDROs in health care settings in Maryland (12, 13). A joint team from the MDH and University of Maryland School of Medicine conducted the survey of 18 ventilator-capable long-term care facilities between 7 March 2023 and 8 June 2023. Sample collection was performed on a single day assigned to each participating facility. Three cultures per patient were obtained on the sample collection day: sputum, perianal, and skin (arm/leg). Sputum samples were obtained during routine respiratory care using a closed tracheal suctioning procedure. Skin (arm/leg) specimens were obtained using Eswabs (Copan Diagnostics), with one swab used per patient to swab both arms and both legs per standardized specimen collection procedure. Patients aged 18 years and older who were either intubated or receiving mechanical ventilation through tracheostomy on the survey day were included. A total of 200 patients had at least 1 sputum, skin, or perianal surveillance culture collected (*n* = 579 individual samples) (Fig. 1).

### Laboratory methods

All isolates were tested for *S. maltophilia* using three microbiological methods in parallel. Furthermore, 0.1 mL of each surveillance culture (sputum, perianal, and skin) was plated via pipette onto CHROMagar Acinetobacter agar and MacConkey agar (Remel) with an imipenem disk and incubated at 35°C–37°C for 24–48 hours. The same sample was used for all three methods.

All samples were also inoculated into brain heart infusion (BHI) broth with an imipenem disk and incubated at 35°C–37°C for 24–48 hours.

After incubation, a sample from all BHI broths, regardless of appearance, was then plated onto MacConkey agar with an imipenem disk and incubated at 35°C–37°C for 24–48 hours.

Red colonies on CHROMagar Acinetobacter agar and lactose non-fermenting organisms on MacConkey agar were identified as possible *S. maltophilia,* and identification was confirmed with the Vitek II system (bioMérieux). Any plates with more than one visible morphology were re-plated to isolate the organisms, decreasing the risk of *Acinetobacter* masking *S. maltophilia* on the same plate. The Vitek II system has previously been shown to accurately identify *S. maltophilia* isolated from clinical samples (14).

Data were stored and analyzed in Microsoft Access.

## RESULTS

All 18 (100%) eligible long-term care facilities with patients receiving mechanical ventilation agreed to participate in the survey, with 200 total patients included in the study. Sputum samples were collected from 97.0% (194/200) of participants, skin samples were collected from 96.5% (193/200) of participants, and perianal samples were collected from 92.5% (185/200) of participants. 21.4% (124/579) of the samples collected grew *S. maltophilia*. Among the 200 patients who had at least one sample collected, 58.5% had at least one culture site positive for *S. maltophilia* (117/200). *S. maltophilia* was isolated from 58.2% (113/194) of sputum samples, 4.1% (8/193) of skin samples, and 1.6% (3/185) of perianal samples (Table 1).

**TABLE 1:** Comparison of *S. maltophilia* across sample sites

Sample site	*S. maltophilia* growth
Sputum	58.2% (113/194)
Skin	4.1% (8/193)
Perianal	1.6% (3/185)
Total	21.4% (124/579)

**Fig 2:**
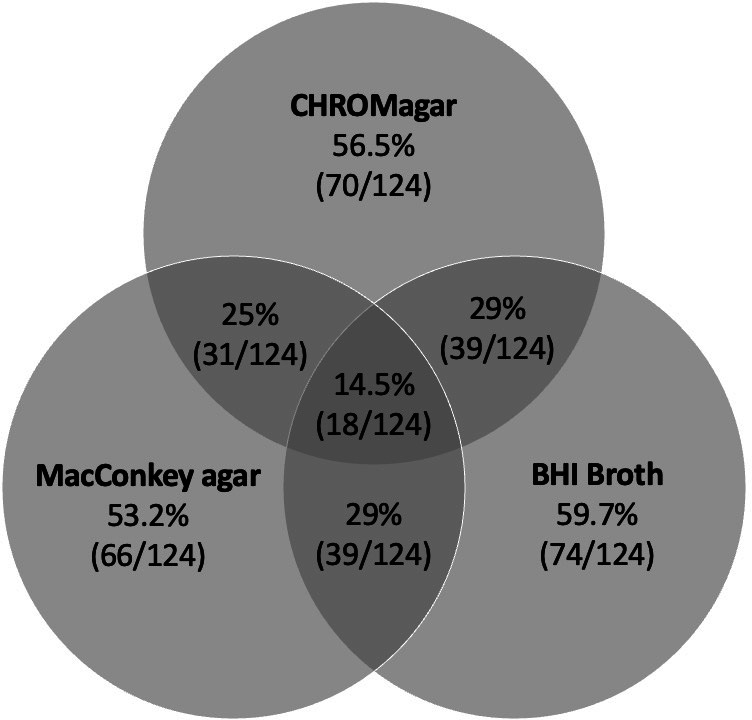
*S. maltophilia* growth across three microbiological methods.

Three microbiological methods were compared for isolating *S. maltophilia*. 56.5% (70/124) of the samples positive for *S. maltophilia* on any method were isolated on the CHROMagar Acinetobacter agar, 53.2% (66/124) were isolated on the MacConkey agar, and 59.7% (74/124) on the BHI broth with an imipenem disk followed by MacConkey agar with a disk (Table 2). 14.5% (18/124) of samples positive for *S. maltophilia* grew on all three methods. Each specimen type (sputum, skin, and perianal) had similar distributions of growth across all three microbiological methods (Table 2). Detection of *S. maltophilia* by only one method occurred in 45.1% (56/124) of samples: 16.1% (20/124) with CHROMagar Acinetobacter agar, 13.7% (17/124) with MacConkey agar, 15.3% (19/124) with BHI broth with an imipenem disk followed by MacConkey agar with a disk (Fig. 2).

**TABLE 2:** Comparison of *S. maltophilia* across sample sites and three microbiological methods

Sample site	Growth on CHROMagar	Growth on MacConkey	Growth on BHI
Sputum	56.6% (64/113)	55.8% (63/113)	62.8% (71/113)
Skin	50.0% (4/8)	25.0% (2/8)	37.5% (3/8)
Perianal	66.7% (2/3)	33.3% (1/3)	0.0% (0/3)
Total	56.5% (70/124)	53.2% (66/124)	59.7% (74/124)

## DISCUSSION

In this state-wide point prevalence survey, we found that more than half of patients receiving mechanical ventilation in Maryland chronic care facilities were colonized with *S. maltophilia. S. maltophilia* was most frequently isolated from sputum cultures compared to skin and perianal cultures. This is consistent with other studies in which respiratory samples were the most common source of *S. maltophilia* colonization (15, 16). While previous studies have documented *S. maltophilia* colonization of the gastrointestinal tract (i.e., in stool samples), very few perianal samples in this study were positive for *S. maltophilia* (17).

To our knowledge, this is the first state-wide point prevalence survey of *S. maltophilia* among patients receiving mechanical ventilation in long-term care facilities. Previous studies have reported the prevalence of colonization among patients with cystic fibrosis (18), COPD (19), or undergoing allogeneic hematopoietic stem cell transplantation (20). Other studies have utilized clinical cultures collected in the context of suspected infection, likely leading to an underestimation of colonization in patients without symptoms of infection (15, 20). Of note, it can be difficult to distinguish between infection and colonization for a low-virulence organism like *S. maltophilia* (20). Furthermore, while the relationship between colonization and infection for *S. maltophilia* remains unclear and updated guidelines focus on the importance of differentiating colonization from infection prior to treatment, colonization is a risk factor for infection in vulnerable patients for many respiratory pathogens (1, 10).

It is documented that *S. maltophilia* grows well under standard laboratory conditions (nutrient-rich media, 37°C) (21), but there are limited studies comparing various microbiological methods for isolating the organism from clinical samples. Previous studies have used vancomycin, imipenem, amphotericin B selective agar, which was shown to be more sensitive for the detection of *S. maltophilia* compared to bacitracin-chocolate agar (22). In this study, we compared three additional methods for isolation of *S. maltophilia*, which were all similar in their sensitivity for isolating *S. maltophilia*. Of note, one culture method alone would have missed a large percentage of samples that were ultimately positive for *S. maltophilia*, emphasizing the possible difficulty of isolating this organism with conventional laboratory methods. It is possible that there was a low level of bacteria present in the samples, which we did not quantify in this study and could have resulted in sporadic growth across the three mediums. Furthermore, there is emerging evidence that not all *S. maltophilia* is inherently resistant to carbapenem antibiotics (23). This would mean that most studies, including our own, are underestimating the prevalence of *S. maltophilia*, as imipenem is frequently used in the process of isolating *S. maltophilia*. Future studies should further investigate methods for isolating this organism, particularly given the growing importance of surveillance.

This study has limitations. First, this point prevalence survey was limited to patients receiving mechanical ventilation in long-term care facilities and thus cannot be widely generalized to other patient populations or care settings. Second, patients receiving mechanical ventilation are a high-risk population that was not compared to a low-risk population such as non-ventilated patients in chronic care facilities or hospitals. Third, genomic sequencing and antimicrobial susceptibility testing were not included in the current study, though genomic sequencing is planned for future studies of these *S. maltophilia* isolates.

Finally, because samples were deidentified, clinical information about the patients from which *S. maltophilia* was isolated, such as whether they subsequently developed a clinical infection or had recent antimicrobial or antiseptic exposure, is unknown. Despite these limitations, this state-wide point prevalence study provides a critical insight into the prevalence of *S. maltophilia* colonization in patients receiving mechanical ventilation in long-term care facilities.

In summary, patients in long-term care facilities who are receiving mechanical ventilation are at high risk of *S. maltophilia* colonization and potential infection. As concern grows over *S. maltophilia* as a multi-drug-resistant pathogen, this patient population should be targeted for surveillance and prevention efforts to better understand the incidence of progression from colonization to infection and to reduce opportunities for transmission in the long-term care setting.
